# MicroRNA-144 attenuates cardiac ischemia/reperfusion injury by targeting FOXO1

**DOI:** 10.3892/etm.2020.8424

**Published:** 2020-01-03

**Authors:** Lusha E, Hong Jiang, Zhibing Lu

Exp Ther Med 17: 2152-2160, 2019; DOI: 10.3892/etm.2019.7161

The first author, Lusha E, contacted the editorial office to explain that the corresponding author was incorrectly assigned for this paper: Instead of Lusha E, the corresponding author on the paper should have been given as Zhibing Lu, the leader and designer of this research project.

Therefore, the information for the corresponding author should have been presented as shown below:

*Correspondence to*: Dr Zhibing Li, Department of Cardiology, Renmin Hospital of Wuhan University, Hubei General Hospital, 238 Jiefang Road, Wuchang, Wuhan, Hubei 430060, P.R. China

E-mail: luzhibingzb@126.com

Lusha E regrets that the corresponding author was not assigned correctly for this paper, and apologizes to the readership for the inconvenience caused.

